# A Bidirectional Relationship Between Hyperuricemia and Metabolic Dysfunction-Associated Fatty Liver Disease

**DOI:** 10.3389/fendo.2022.821689

**Published:** 2022-02-16

**Authors:** Chengzhang Yang, Qianjin He, Ze Chen, Juan-Juan Qin, Fang Lei, Ye-Mao Liu, Weifang Liu, Ming-Ming Chen, Tao Sun, Qian Zhu, Yonglin Wu, Ming Zhuo, Jingjing Cai, Weiming Mao, Hongliang Li

**Affiliations:** ^1^ Department of Cardiology, Renmin Hospital of Wuhan University, Wuhan, China; ^2^ Institute of Model Animal, Wuhan University, Wuhan, China; ^3^ Department of Hepatobiliary Surgery, Huanggang Central Hospital, Huanggang, China; ^4^ Huanggang Institute of Translational Medicine, Huanggang Central Hospital, Huanggang, China; ^5^ Department of Cardiology, Zhongnan Hospital of Wuhan University, Wuhan, China; ^6^ School of Basic Medical Science, Wuhan University, Wuhan, China; ^7^ Department of Cardiology, Huanggang Central Hospital, Huanggang, China; ^8^ Department of Pharmacy, Guangdong Provincial People’s Hospital, Guangdong Academy of Medical Sciences, Guangzhou, China; ^9^ Department of Anesthesiology, The First Affiliated Hospital of Gannan Medical College, Ganzhou, China; ^10^ First Clinical College, Medical College of Soochow University, Suzhou, China; ^11^ Department of Cardiology, The Third Xiangya Hospital, Central South University, Changsha, China; ^12^ Department of General Surgery, Huanggang Central Hospital, Huanggang, China

**Keywords:** hyperuricemia, metabolic dysfunction-associated fatty liver disease, cross-lagged path analysis, metabolism, nonalcoholic fatty liver disease

## Abstract

**Background and aims:**

Metabolic dysfunction-associated fatty liver disease (MAFLD) is a newly emerged term that is suggested to better reflect the pathogenesis of nonalcoholic fatty liver disease (NAFLD); however, the association between hyperuricemia and MAFLD has not been explored in the Chinese population. Meantime, this study also examined the temporal relationship between the two entities in a longitudinal cohort.

**Methods:**

We conducted a retrospective cross-sectional study including 1,587,962 individuals from 19 health check-up centers in China from 2009-2017 and a longitudinal study with 16,112 individuals. A logistic regression model was applied to determine the association between hyperuricemia and MAFLD in a cross-sectional study. The Cox regression model was used to explore the association between hyperuricemia at baseline and subsequent onset of MAFLD or the association between the presence of MAFLD at baseline and the subsequent incidence of hyperuricemia. The cross-lagged analysis was applied to exam the temporal relationship between hyperuricemia and MAFLD.

**Results:**

In the cross-sectional study, hyperuricemia showed a strong positive association with MAFLD after controlled potential confounders. In the longitudinal cohorts, hyperuricemia at baseline was associated with the new-onset of MAFLD, with a hazard ratio (HR) of 1.765 (95% CI: 1.512, 2.060). Interestingly, baseline MAFLD was also associated with the subsequent incidence of hyperuricemia, with an HR of 1.245 (95% CI: 1.106, 1.400). The cross-lagged path analysis revealed a bidirectional relationship between hyperuricemia and MAFLD.

**Conclusions:**

The results suggested that hyperuricemia and MAFLD form a vicious cycle, resulting in more deterioration of metabolic status.

## Introduction

Metabolic dysfunction-associated fatty liver disease (MAFLD), formerly known as nonalcoholic fatty liver disease (NAFLD), is a new term that focuses more on systemic metabolic dysfunction (1). In the past, the diagnosis of NAFLD was a diagnosis of exclusion (2); however, with advances in the understanding of NAFLD pathogenesis, it has been found that NAFLD is derived from a variety of potential states of metabolic dysfunction and has complex pathophysiological characteristics, which indicates that the exclusion criteria can no longer meet the current diagnostic requirements for the disease. Therefore, in the recent international expert consensus, “MAFLD” is perceived as a standalone disease related to known metabolic dysfunction and has a specific positive diagnosis (1). This change will inevitably lead to considerable differences between the MAFLD and the NAFLD populations. MAFLD has a higher prevalence rate and a higher risk of cardiovascular diseases, which has brought an enormous health and economic burden to the world (3). Uric acid is the final oxidation product of hypoxanthine and xanthine catabolism. Although many studies have confirmed that uric acid is a risk factor for metabolic syndrome, cardiovascular disease, and NAFLD (4, 5), the relationship between hyperuricemia and MAFLD is still unclear due to the marked difference between the NAFLD and MAFLD populations.

Therefore, we used the largest cross-sectional database based on Chinese health check-up centers to analyze the association between hyperuricemia and MAFLD. Furthermore, this study also examined the temporal relationship between the two entities in a longitudinal cohort among this population.

## Materials and Methods

### Study Population

A total of 1,593,135 participants from 19 health management centers between January 2009 and December 2017 in China were included in our study. Participants who 1) were younger than 18 years old; 2) had liver cirrhosis, liver cancer, or a history of liver surgery; 3) had kidney-related diseases that may affect uric acid metabolism (nephrotic syndrome, kidney tumors, and renal failure); 4) treating with uric acid-lowering drugs; and 5) lacked sufficient information for making MAFLD diagnoses were excluded. Finally, 1,587,962 individuals were included in the cross-sectional study.

**Figure 1:**
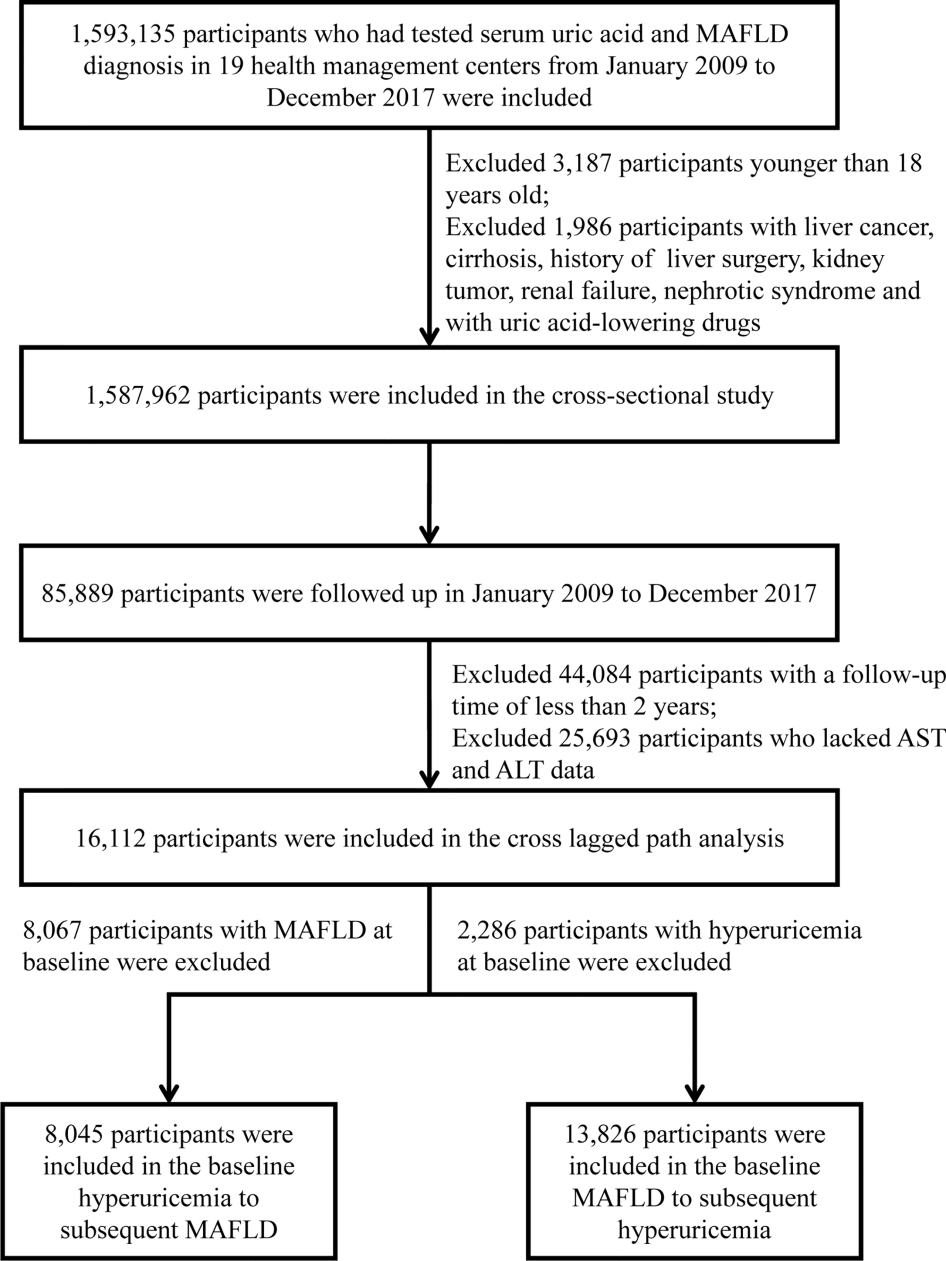
The flow chart of the participants’ enrollment. MAFLD, metabolic dysfunction-associated fatty liver disease; AST, aspartate aminotransferase; ALT, alanine aminotransferase.

Of the 1,587,962 individuals, 85,889 had undergone follow-up. Among these participants, 16,112 individuals who had at least 2 years of follow-up had repeated measurements of serum uric acid level, imaging examinations for liver fat (abdominal ultrasound, computed tomography (CT), or magnetic resonance imaging (MRI)) and had complete hepatic steatosis index (HSI) information were considered part of a longitudinal cohort. To explore the association between hyperuricemia at baseline and subsequent new onset of MAFLD, we excluded participants with MAFLD at baseline to constitute Cohort 1, including 8,045 individuals. Conversely, to investigate the association between the presence of MAFLD at baseline and the subsequent incidence of hyperuricemia, we excluded individuals with hyperuricemia at baseline to form Cohort 2, including 13,826 individuals. The flow chart of participant selection is shown in **Figure 1**.

This study was approved by the central ethics board of Renmin Hospital of Wuhan University and followed by acceptance with the ethics center in each collaborating hospital. Ethics committees granted a waiver of the requirement for documentation of informed consent for just analyzing existing data after anonymization without individual identification.

### Anthropometric and Laboratory Data

All participants had undergone comprehensive anthropometric measurements and clinical examinations by professional and experienced medical teams in each hospital. Anthropometric measurements, including height, weight, and waist circumference (WC), were measured in subjects wearing light clothes without shoes. The calculation method of body mass index (BMI) was weight (kg)/height square (m^2^). Systolic blood pressure (SBP) and diastolic blood pressure (DBP) were measured by mercury sphygmomanometers or electronic sphygmomanometers after individuals rested in a seated position for a minimum of 5 min. The measurement was carried out three times in succession at intervals of 1 minute, and the average value of the measurement results was taken. Fasting blood samples were drawn from an antecubital vein after participants had fasted over 12 h. We measured fasting blood glucose (FBG), total cholesterol (TC), triglycerides (TG), low-density lipoprotein cholesterol (LDL-C), high-density lipoprotein cholesterol (HDL-C), alanine aminotransferase (ALT), aspartate transaminase (AST), blood urea nitrogen (BUN) and serum creatinine (SCR) concentrations with an automatic biochemical analyzer 2 hours after sample collection. All checkup centers included in our study were applied identical methodology, namely, urease peroxidase coupling colorimetry, to measure serum uric acid based on the Trinder reaction.

### Diagnostic Criteria

MAFLD was defined by evidence of hepatic steatosis on abdominal ultrasound, CT, or MRI with the presence of one of the following three criteria: overweight or obesity (defined as BMI ≥ 23 kg/m^2^ in Asians), type 2 diabetes mellitus (T2DM), or metabolic dysregulation (1). Metabolic dysregulation was defined by the presence of at least two of the following metabolic risk abnormalities: 1) WC ≥ 90 cm for men and 80 cm for women; 2) blood pressure ≥ 130/85 mmHg or on specific drug treatment; 3) plasma TG ≥ 1.70 mmol/L or on specific drug treatment; 4) plasma HDL-C < 1.0 mmol/L for men and < 1.3 mmol/L for women or on specific drug treatment; 5) prediabetes (i.e., fasting glucose levels 5.6 to 6.9 mmol/L, or 2-h post-load glucose levels 7.8 to 11.0 mmol or glycosylated hemoglobin A1c (HbA1c) 5.7% to 6.4%).

The diagnosis of hepatic steatosis on ultrasound was based on hepatorenal echo contrast, liver parenchymal brightness, deep attenuation, and vascular blurring. The diagnosis of hepatic steatosis on CT examination was based on two reference standards: a liver-spleen attenuation difference of greater than 10 HU and the absolute attenuation of the liver less than 40 HU. The diagnosis of hepatic steatosis on MRI examination was based on the fact that the reversed-phase image shows signal loss relative to the positive phase in phase interference imaging. Over 30% of steatosis was considered a fatty liver disease. The hepatic steatosis index (HSI) was applied to quantify the degree of steatosis of MAFLD. HSI=8*ALT/AST+BMI (+2 if diabetes, +2 if female) (6).

Based on epidemiological definitions, hyperuricemia was identified as a serum uric acid level > 420 μmol/L (7.0 mg/dL) in males and > 360 μmol/L (6.0 mg/dL) in females (7). Hypertension was defined as personal medical history, use of antihypertensive drugs, and/or SBP ≥ 140 mmHg and/or DBP ≥ 90 mmHg (8). Diabetes was defined as fasting blood glucose ≥ 7.0 mmol/L, 2 h postprandial glucose ≥ 11.1 mmol/L, personal history or use of hypoglycemic drugs (9).

### Statistical Analysis

Categorical variables were presented as frequencies and percentages. Nonnormally distributed continuous variables were expressed as the median and interquartile range (IQR). When comparing differences between groups, the Kruskal–Wallis test was used for continuous variables, and the χ2 test or Fisher’s exact test was used for categorical variables. The nonparameter imputation method missForest was conducted to deal with missing data, and the estimated imputation error was 6%. Logistic progression models were applied to examine the association between hyperuricemia and MAFLD. Cox proportional hazards models were used to evaluate the association between hyperuricemia at baseline and the incidence of MAFLD or the association between baseline MAFLD and the subsequent incidence of hyperuricemia. Statistical significance was considered as two-sided *P* < 0.05. All data were analyzed using R-4.0.2 (R Foundation for Statistical Computing, Vienna, Austria), SPSS Statistics (version 25.0, IBM, Armonk, NY, USA), and IBM Amos (version 23.0, SPSS Inc, Chicago, IL, USA).

### Cross-Lagged Path Analysis

**Figure 2:**
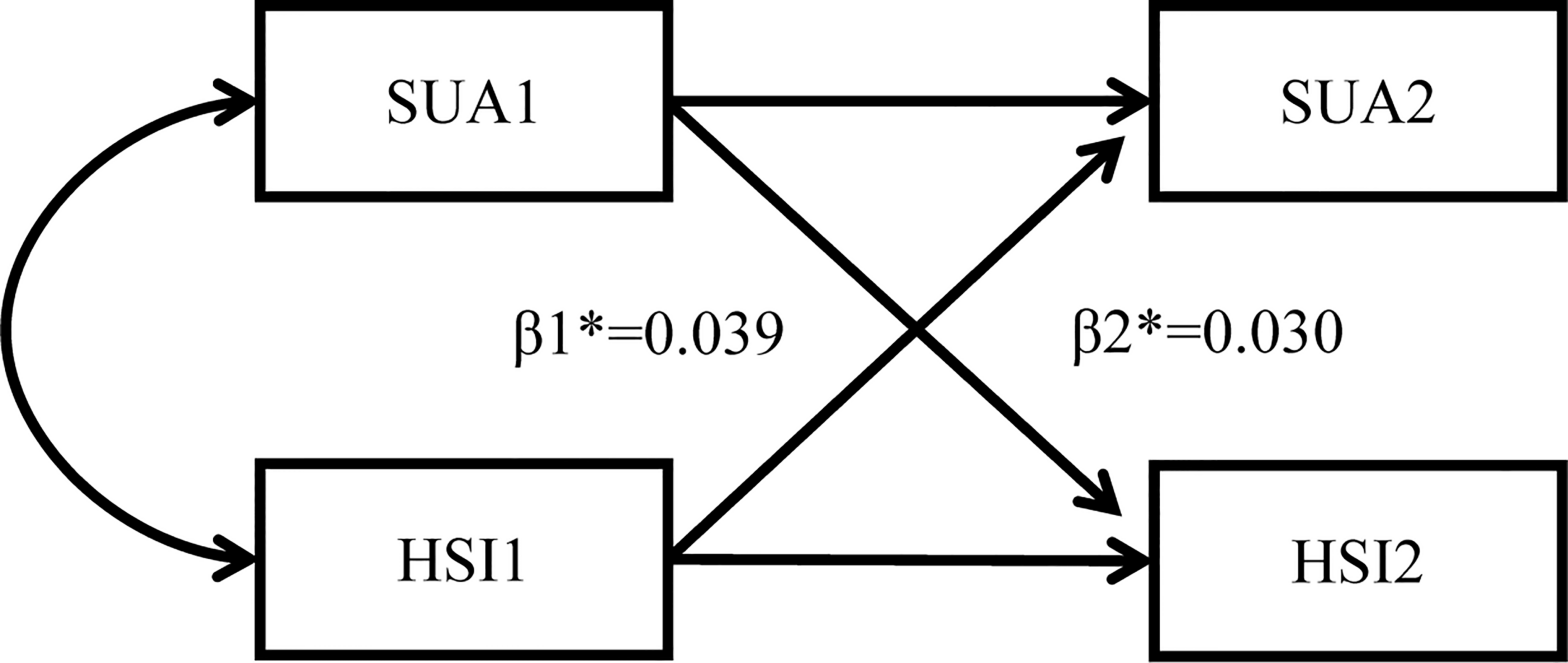
Cross-lagged path analysis pattern diagram. SUA, serum uric acid; HSI, hepatic steatosis index *: β1, baseline hyperuricemia to subsequent MAFLD; β2, baseline MAFLD to subsequent hyperuricemia; MAFLD, metabolic dysfunction-associated fatty liver disease.

To explore the temporal relationship between hyperuricemia and MAFLD, the cross-lagged path analysis was applied (10, 11). Serum uric acid and liver steatosis were measured repeatedly at two time points. Before the cross-lagged analysis, both baseline and follow-up values were controlled by residual regression analyses and then standardized by Z-transformation (mean = 0, SD = 1). Baseline and follow-up variables were adjusted *via* regression residual analyses before cross-lagged analysis. Adjusted confounding variables included age, sex, ALT, FBG, SBP, LDL-C, BUN, and TG. In the cross-lagged panel analysis, the temporal relationship between hyperuricemia and MAFLD can be determined by comparing standardized path coefficients β1 (baseline hyperuricemia to subsequent MAFLD) and β2 (baseline MAFLD to subsequent hyperuricemia). Fisher’s Z test was used to compare the difference between β1 and β2 (10). The root mean square residual (RMR) and comparative fitness index (CFI) were used to evaluate the fit of the model. If the model fitting parameters RMR<0.05 and CFI>0.90, it proves that the fit is good (11). The cross-lagged path analysis pattern diagram is presented in **Figure 2**.

### Sensitivity Analysis

To assess the robustness of the bidirectional association between hyperuricemia and MAFLD and eliminate the influence of confounding variables, we performed two sensitivity analyses. First, we further adjusted SCR and HDL-C based on age, sex, LDL-C, FBG, ALT, BUN, SBP, and TG in the sensitivity analysis 1. Second, we further performed sensitivity analysis 2 using a population only diagnosed by ultrasound.

## Results

### Anthropometric and Laboratory Characteristics of Subjects in the Cross-Sectional Study

We included 1,587,962 participants in the cross-sectional study. The median age of the overall population was 46.00 years (IQR, 36.00, 55.00), and males accounted for 60.34%. Baseline characteristics of individuals are shown in **Table 1**. In the overall population, 40.05% of participants had MAFLD, and 13.78% of individuals had hyperuricemia. The median age of MAFLD participants was 48.00 years (IQR, 39.00, 56.00), which was older than that of the participants without MAFLD, with a median age of 44.00 years (IQR, 34.00, 54.00) (*P* < 0.001). The median age of participants with hyperuricemia was 44.00 years (IQR, 35.00, 54.00), which was younger than the population without hyperuricemia, with a median age of 46.00 years (IQR, 36.00, 55.00). Males accounted for 72.66% of the population with MAFLD and 87.23% of the population with hyperuricemia.

**Table 1:** Baseline characteristics of 1,587,962 participants stratified by MAFLD and hyperuricemia in the sectional study.

	Overall(n=1,587,962)	Non-MAFLD(n=951,985)	MAFLD(n=635,977)	*P*-value^*^	Non-hyperuricemia(n=1,369,166)	Hyperuricemia(n=218,796)	*P*-value^*^
Age, median (IQR) years	46.00 [36.00, 55.00]	44.00 [34.00, 54.00]	48.00 [39.00, 56.00]	<0.001	46.00 [36.00, 55.00]	44.00 [35.00, 54.00]	<0.001
Male sex, n(%)	958184 (60.34)	496056 (52.11)	462128(72.66)	<0.001	767337(56.04)	190847(87.23)	<0.001
FBG (mmol/L, median (IQR))	5.16 [4.79, 5.60]	5.04 [4.70, 5.40]	5.40 [4.97, 5.98]	<0.001	5.14 [4.78, 5.60]	5.25 [4.85, 5.73]	<0.001
LDL-C (mmol/L, median (IQR))	2.72 [2.21, 3.28]	2.60 [2.12, 3.14]	2.91 [2.38, 3.46]	<0.001	2.70 [2.20, 3.26]	2.84 [2.31, 3.39]	<0.001
HDL-C (mmol/L, median (IQR))	1.30 [1.10, 1.55]	1.40 [1.18, 1.65]	1.16 [1.00, 1.36]	<0.001	1.33 [1.12, 1.58]	1.14 [0.98, 1.33]	<0.001
TC (mmol/L, median (IQR))	4.72 [4.14, 5.35]	4.58 [4.04, 5.19]	4.93 [4.34, 5.57]	<0.001	4.69 [4.12, 5.32]	4.92 [4.33, 5.56]	<0.001
TG (mmol/L, median (IQR))	1.31 [0.89, 1.99]	1.05 [0.76, 1.51]	1.83 [1.30, 2.65]	<0.001	1.24 [0.85, 1.85]	1.92 [1.31, 2.86]	<0.001
WC (cm, median (IQR))	83.00 [75.00, 90.00]	78.00 [71.00, 84.00]	91.00 [86.00, 96.00]	<0.001	82.00 [74.00, 89.00]	90.00 [84.00, 96.00]	<0.001
BMI (kg/m^2^, median (IQR))	24.53 [22.43, 26.69]	23.20 [21.09, 24.96]	26.51 [24.87, 28.40]	<0.001	24.25 [22.10, 26.37]	26.17 [24.32, 28.28]	<0.001
ALT (U/L, median (IQR))	21.00 [15.00, 31.00]	18.00 [13.00, 25.00]	27.90 [19.64, 40.00]	<0.001	20.00 [14.00, 29.00]	29.00 [20.00, 42.10]	<0.001
AST (U/L, median (IQR))	21.40 [17.70, 26.40]	20.30 [17.00, 25.00]	23.00 [19.00, 29.00]	<0.001	21.00 [17.00, 26.00]	24.00 [20.00, 31.00]	<0.001
BUN (mmol/L, median (IQR))	4.63 [3.87, 5.51]	4.51 [3.76, 5.40]	4.80 [4.04, 5.68]	<0.001	4.60 [3.81, 5.48]	4.91 [4.17, 5.80]	<0.001
SUA (mmol/L, median (IQR))	319.40 [258.20, 383.00]	291.80 [237.10, 352.00]	359.60 [303.10, 418.40]	<0.001	303.00 [249.00, 355.00]	465.60 [440.70, 504.00]	<0.001
SCR (umol/L, median (IQR))	72.00 [60.00, 83.00]	69.00 [57.40, 80.50]	75.20 [65.00, 86.00]	<0.001	70.00 [58.50, 81.00]	82.00 [73.00, 92.00]	<0.001
SBP (mmHg, median (IQR))	122.00 [110.00, 135.00]	119.00 [109.00, 130.00]	130.00 [119.00, 140.00]	<0.001	120.00 [110.00, 134.00]	128.00 [118.00, 140.00]	<0.001
DBP (mmHg, median (IQR))	77.00 [70.00, 85.00]	73.00 [67.00, 80.00]	81.00 [74.00, 90.00]	<0.001	76.00 [69.00, 84.00]	80.00 [73.00, 90.00]	<0.001
BMI≥23, n(%)	1117303 (70.36)	509863 (53.56)	607440 (95.51)	<0.001	922215 (67.36)	195088 (89.16)	<0.001
Hypertension, n(%)	432501 (27.33)	176297 (18.58)	256204 (40.44)	<0.001	349293 (25.60)	83208 (38.16)	<0.001
Diabetes, n(%)	120285 (7.57)	38503 (4.04)	81782 (12.86)	<0.001	104992 (7.67)	15293 (6.99)	<0.001
Hyperuricemia, n(%)	218796 (13.78)	74160 (7.79)	144636 (22.74)	<0.001	–	–	–
MAFLD, n(%)	635977 (40.05)	–	–	–	491341 (35.89)	144636 (66.11)	<0.001

FBG, fasting blood glucose; LDL-C, low-density lipoprotein cholesterol; HDL-C, high-density lipoprotein cholesterol; TC, total cholesterol; TG, triglycerides; WC, waist circumference; BMI, body mass index; ALT, alanine aminotransferase; AST, aspartate transaminase; BUN, blood urea nitrogen; SUA, serum uric acid; SCR, serum creatinine; SBP, systolic blood pressure; DBP, diastolic blood pressure; MAFLD, metabolic dysfunction-associated fatty liver disease.

*: P-value was calculated by Kruskal-Wallis test for continuous variables, as well as the χ2 test or Fisher’s exact test for categorical variables.

The population with MAFLD and hyperuricemia had higher BMI, WC, LDL-C, TC, TG, ALT, AST, BUN, SCR, SBP, and DBP than those in the non-MAFLD and non-hyperuricemia groups. Notably, the SUA levels were significantly higher in the subjects with MAFLD than in the non-MAFLD group. Similarly, the population with hyperuricemia had a higher prevalence of MAFLD than those without hyperuricemia (**Table 1**).

### Association Between Hyperuricemia and the Prevalence of MAFLD in the Cross-Sectional Analysis

Logistic regression analysis was performed to identify the association between hyperuricemia and MAFLD. In the unadjusted model, hyperuricemia was significantly associated with MAFLD, with an odds ratio (OR) of 3.484 (95% confidence interval [CI]: 3.451, 3.518) (*P* < 0.001). After adjusting for age, sex, LDL-C, FBG, ALT, BUN, SBP, and TG, the relationship between hyperuricemia and MAFLD remained statistically significant, with an OR of 2.048 (95% CI: 2.026, 2.070) (*P* < 0.001) (**Table 2**).

**Table 2:** The association between hyperuricemia and MAFLD by the logistic regression in the cross-sectional study.

	OR (95%CI)	P-value
Crude	3.484 (3.451, 3.518)	<0.001
Model 1	2.931 (2.902, 2.960)	<0.001
Model 2	2.048 (2.026, 2.070)	<0.001

MAFLD, metabolic dysfunction-associated fatty liver disease.

Model 1: adjusted for age and sex.

Model 2: adjusted for age, sex, low-density lipoprotein cholesterol (LDL-C), fasting blood glucose (FBG), alanine aminotransferase (ALT), blood urea nitrogen (BUN), systolic blood pressure (SBP), triglycerides (TG).

### Anthropometric and Laboratory Characteristics of Subjects in the Longitudinal Cohort Study

We implemented two longitudinal cohort studies to confirm the temporal relationship between hyperuricemia and MAFLD. In the Cohort 1, 1,634 participants developed MAFLD during the follow-up among 8,045 participants without MAFLD at baseline. The median age of the population developed MAFLD was 47.00 years (IQR, 39.00, 55.00), which was older than that of the non-MAFLD group, with a median age of 45.00 years (IQR, 36.00, 55.00). The population that developed MAFLD had a greater proportion of males than those who did not develop MAFLD (69.22% vs. 53.21%). The population that developed MAFLD also had a higher proportion of hypertension and diabetes and had a higher BMI, LDL-C, TC, TG, WC, ALT, BUN, SCR, SBP, DBP, and BUN but lower HDL-C. Notably, a significantly higher proportion of participants with hyperuricemia was observed in the subjects who developed MAFLD than in the non-MAFLD group (12.67% vs. 5.41%). The baseline characteristics of individuals stratified by MAFLD are described in **Supplementary Table 1**.

In the Cohort 2, 11.00% of participants (n=1,516) developed hyperuricemia during the follow-up among 13,826 participants without hyperuricemia at baseline. Individuals who developed hyperuricemia were younger than those who did not, with median ages of 46.00 years (IQR, 36.00, 55.00) and 47.00 years (IQR, 40.00, 55.00), respectively. Males accounted for a considerably higher proportion in the group that developed hyperuricemia than in the non-hyperuricemia group (87.73% vs. 61.10%). The hyperuricemia group also had a higher BMI, WC, ALT, AST, BUN, SCR, SBP, and DBP but lower LDL-C and HDL-C. Notably, the proportion of MAFLD participants in the hyperuricemia group was significantly greater than that in the non-hyperuricemia group (58.25% vs. 44.29%). The baseline characteristics stratified by hyperuricemia are shown in **Supplementary Table 2**.

### Association Between Baseline Hyperuricemia and the Development of MAFLD and Between Baseline MAFLD and the Development of Hyperuricemia in the Longitudinal Cohort

In the unadjusted analysis of hyperuricemia at baseline and the subsequent development of MAFLD, participants with hyperuricemia at baseline showed a higher risk of developing MAFLD during follow-up, with a hazard ratio (HR) of 2.670 (95% CI: 2.307, 3.090). After adjusting for age, sex, LDL-C, FBG, ALT, BUN, SBP, TG, and insulin resistance, baseline hyperuricemia was still significantly associated with the development of MAFLD, with an HR of 1.765 (95% CI: 1.512, 2.060) (*P* < 0.001) (**Table 3**).

**Table 3:** The association between hyperuricemia and MAFLD by the Cox regression in the longitudinal cohort study.

Baseline hyperuricemia to subsequent MAFLD			Baseline MAFLD to subsequent hyperuricemia		
	HR(95%CI)	*P*-value		HR(95%CI)	*P*-value
Crude	2.670 (2.307,3.090)	<0.001	Crude	1.765 (1.594,1.955)	<0.001
Model 1	2.192 (1.887,2.546)	<0.001	Model 1	1.395 (1.257,1.549)	<0.001
Model 2	1.766 (1.512,2.061)	<0.001	Model 2	1.255 (1.116,1.412)	<0.001
Model 3	1.765 (1.512,2.060)	<0.001	Model 3	1.245 (1.106,1.400)	<0.001

MAFLD, metabolic dysfunction-associated fatty liver disease.

Model 1: adjusted for age and sex.

Model 2: adjusted for age, sex, low-density lipoprotein cholesterol (LDL-C), fasting blood glucose (FBG), alanine aminotransferase (ALT), blood urea nitrogen (BUN), systolic blood pressure (SBP), triglycerides (TG).

Model 3: adjusted for age, sex, low-density lipoprotein cholesterol (LDL-C), fasting blood glucose (FBG), alanine aminotransferase (ALT), blood urea nitrogen (BUN), systolic blood pressure (SBP), triglycerides (TG), insulin resistance.

When analysis the association between MAFLD at baseline and the later occurrence of hyperuricemia, the crude model indicated that MAFLD at baseline was significantly associated with the occurrence of hyperuricemia, with an HR of 1.765 (95% CI: 1.594, 1.955) (*P* < 0.001). This association remained significant after adjusting for confounding variables as mentioned above, with an HR of 1.245 (95% CI: 1.106, 1.400) (**Table 3**).

### Cross-Lagged Path Analysis Between Hyperuricemia and MAFLD

The temporal relationship between hyperuricemia and MAFLD was detected by cross-lagged path analysis. The standardized regression coefficient from baseline hyperuricemia to subsequent development of MAFLD was β1 = 0.039 (95% CI: 0.026, 0.051), and the standardized regression coefficient from baseline MAFLD to subsequent development of hyperuricemia was β2 = 0.030 (95% CI: 0.017, 0.043) after adjusting for age, sex, LDL-C, FBG, ALT, BUN, SBP, TG, and insulin resistance. In addition, Fisher’s z test indicated no significant difference between β1 and β2 (*P* = 0.418), which indicated that hyperuricemia and MAFLD affect each other and that there was no prior relationship. The RMR and CFI were 0.022<0.05 and 0.988>0.9, respectively, indicating that the model fit was good (**Table 4**).

**Table 4:** The cross-lagged path analysis for the temporal association between hyperuricemia and MAFLD.

	β1(hyperuricemia to subsequent MAFLD)	*P*-value	β2(MAFLD to subsequent hyperuricemia)	*P*-value	RMR	CFI
Crude	0.058 (0.046, 0.071)	<0.001	0.028 (0.016, 0.040)	<0.001	0.018	0.992
Model 1	0.040 (0.028, 0.051)	<0.001	0.027 (0.013, 0.040)	<0.001	0.020	0.990
Model 2	0.039 (0.027, 0.051)	<0.001	0.032 (0.019, 0.045)	<0.001	0.021	0.988
Model 3	0.039 (0.026, 0.051)	<0.001	0.030 (0.017, 0.043)	<0.001	0.022	0.988

MAFLD, metabolic dysfunction-associated fatty liver disease; RMR, root mean square residual; CFI, comparative fitness index.

Model 1: adjusted for age, sex.

Model 2: adjusted for age, sex, low-density lipoprotein cholesterol (LDL-C), fasting blood glucose (FBG), alanine aminotransferase (ALT), blood urea nitrogen (BUN), systolic blood pressure (SBP), triglycerides (TG).

Model 3: adjusted for age, sex, low-density lipoprotein cholesterol (LDL-C), fasting blood glucose (FBG), alanine aminotransferase (ALT), blood urea nitrogen (BUN), systolic blood pressure (SBP), triglycerides (TG), insulin resistance.

### Sensitivity Analysis

In the sensitivity test 1, in addition to age, sex, LDL-C, FBG, ALT, BUN, SBP, and TG, we further adjusted SCR and HDL-C as confounders, the standardized regression coefficient β1 and β2 were 0.035 (95% CI: 0.023, 0.047) and 0.025 (95% CI: 0.012, 0.038), respectively. No significant difference existed between β1 and β2 (*P* = 0.368) examined by Fisher’s z test, which indicated that hyperuricemia and MAFLD were still bidirectional relationships after controlling for more confounding variables (**Supplementary Table 3**). In the sensitivity analysis 2, we performed the cross-lagged analysis in population had abdominal ultrasound examination. The result was found to be consistent with that in the overall population (**Supplementary Table 4** and **Supplementary Table 5**).

## Discussion

Our study was the first to provide evidence on the bidirectional relationship between hyperuricemia and MAFLD in a large-sample cohort from health check-up centers. First, we used the largest cross-sectional database in China and found that hyperuricemia and MAFLD are closely related. Second, the results of the longitudinal cohort 1 study showed that hyperuricemia was associated with a new occurrence of MAFLD. Similarly, MAFLD was also related to the incidence of hyperuricemia in longitudinal cohort 2. Finally, the cross-lagged path analysis also proved the two-way relationship between hyperuricemia and MAFLD, indicating that hyperuricemia and MAFLD have intimate mutual effects during disease progression. MAFLD patients need to monitor serum uric acid levels to prevent the occurrence of hyperuricemia, and ameliorating serum uric acid levels in hyperuricemia patients may prevent subsequent MAFLD.

Recently, a change in nomenclature from NAFLD to MAFLD has been proposed, with the purpose of overcoming the limitations of NAFLD definition (1). The NAFLD and MAFLD populations overlapped but were not identical. A study has shown that the MAFLD population had a higher body mass index (BMI), proportion of metabolic comorbidities and ALT levels than those with NAFLD. Moreover, MAFLD individuals have increased the overall risk for total mortality by even greater magnitude than the NAFLD population (12). Previously, the association between serum uric acid level and NAFLD has been demonstrated in cross-sectional studies and longitudinal studies (4, 13). The MAFLD population is distinct from the NAFLD population, and further investigation into the association between hyperuricemia and MAFLD would be meaningful for formulating preventive strategies for these two diseases.

Previous cross-sectional studies on Asian populations showed that both hyperuricemia as a dichotomized variable and uric acid levels as a continuous variable were closely associated with NAFLD (4, 14). A prospective observational study further revealed that elevated serum uric acid levels independently predict an increased risk for incident NAFLD in the Chinese population (15). Because populations with NAFLD and MAFLD share many metabolic traits, our observations in MAFLD were consistent with the findings in the NAFLD population. Our study is the first study with the largest cross-sectional database and showed the close association between hyperuricemia and MAFLD independent of other traditional metabolic risk factors. Moreover, using a longitudinal cohort, we also revealed that hyperuricemia at baseline was associated with incident MAFLD. Given the higher relevance to systemic metabolic dysfunction in MAFLD, we continuously explored the temporal relationship between baseline MAFLD and the subsequent development of hyperuricemia in the cohort. The temporal association of NAFLD with the incidence of hyperuricemia has been controversial in previous studies. A 7-year prospective study showed that NAFLD significantly increases the risk of subsequent incident hyperuricemia in a cohort of 5541 baseline hyperuricemia-free individuals (13). However, this observation was not observed in other NAFLD cohorts, only a unidirectional relationship from hyperuricemia to NAFLD incidence was established (11). In our cohort, we observed that MAFLD increased the risk of subsequent hyperuricemia. Summarizing the abovementioned results suggested that hyperuricemia and MAFLD were a bidirectional relationship and formed a vicious cycle in the development of systematic metabolic disorders. Similar to many metabolic disorders, it occurs as a collection of disorders and increases your risk of developing more complex metabolic disorders or cardiovascular disease (16). Individuals with certain metabolic diseases should be evaluated and intervened systematically to reduce the overall risk of cardiovascular diseases and mortality.

Some mechanisms explain the bidirectional relationship between hyperuricemia and MAFLD. Hyperuricemia and MAFLD can affect each other through direct effects. An excessive uric acid level can directly induce hepatic steatosis through NOD-like receptor family pyrin domain containing 3 (NLRP3) inflammasome-dependent mechanism (17). Increased uric acid also activated nicotinamide adenine dinucleotide phosphate (NADPH) oxidase, leading to reactive oxygen species (ROS) formation in the mitochondria, which led to liver fat production. ROS accumulation also induces a stress cascade in the endoplasmic reticulum, which subsequently increases the expression of adipogenic genes and the production of triglycerides (18). NAFLD can, in turn, directly lead to hyperuricemia. Xu et al.’s study indicated that expression and activity of xanthine oxidase, a rate-limiting enzyme that catalyzed uric acid production, were significantly increased in cellular and mouse models of NAFLD, which resulted in significantly elevated serum uric acid levels (13).

Apart from the abovementioned direct effects between hyperuricemia and MAFLD, insulin resistance (IR) may amplify this interaction. On the one hand, hyperinsulinemia will increase uric acid synthesis and reduce uric acid excretion (4). On the other hand, Rinaldi et al. illustrated that hyperinsulinemia also induced an accumulation of lipid in the liver. Excessively accumulated fatty acids further decrease insulin sensitivity in the liver (19). Meantime, IR amplifies the uric acid-induced NLRP3 inflammasome-dependent mechanism in hepatocytes and increases the inflammatory response in the liver (17). These mentioned metabolic disturbances form a vicious cycle and lead to vascular endothelial cell damage and smooth muscle cell proliferation (20). This mechanism partly explains the elevated cardiovascular risk in NAFLD patients. Although these potential mechanisms suggested that the bidirectional relationship between hyperuricemia and MAFLD may be through direct or indirect effects mediated by insulin resistance, more high-quality data from clinical and basic studies are needed to inform the current debate.

### Limitations

The study has certain limitations that merit attention. First, there is an inherent bias in exploring the causal relationship between hyperuricemia and MAFLD because of the retrospective design, and more definitive validation in prospective studies is needed. Second, because MAFLD is mainly diagnosed by ultrasound, the severity of MAFLD-associated hepatitis cannot be determined, and mild steatosis may not be detected. Third, we chose the presence of at least two of five metabolic risk abnormalities as the definition of metabolic dysregulation because of limited data on insulin resistance and high-sensitivity C-reactive protein, which will reduce the detection rate of MAFLD. Fourth, the use of HSI as a surrogate indicator for MAFLD is somewhat biased, but there is no recognized surrogate indicator yet. Fifth, dietary factors that lead to hyperuricemia including eating purine-rich foods, were not considered. Sixth, since the number of individuals with insulin resistance indicators is relatively small in the cross-sectional population, we only adjusted insulin resistance as a confounder in the longitudinal cohort.

## Conclusion

This study showed a bidirectional relationship between hyperuricemia and MAFLD, which suggested that MAFLD and hyperuricemia may form a vicious cycle and lead to a more deteriorated metabolic status. Further prospective studies are needed to elucidate the interaction between these two entities.

## Data Availability Statement

The original contributions presented in the study are included in the article/**Supplementary Material**. Further inquiries can be directed to the corresponding authors.

## Author Contributions

In our research, CY and QH designed the study, analyzed data, and wrote the manuscript. ZC, WL, M-MC, TS, QZ, MZ, and YW collected and contributed to data analysis. FL, JQ, and Y-ML wrote codes for data analysis. JC revised the manuscript. HL and WM contributed equally, designed the project, edited the manuscript, and supervised the study. All authors have approved the final version of this paper.

## Funding

This work was supported by grants from the National Science Foundation of China (82000299, 81630011, 81870171), the Hubei Science and Technology Support Project (2019BFC582, 2018BEC473), the Medical flight plan of Wuhan University (TFJH2018006) and the Henan Charity Federation Hepatobiliary Fund (GDXZ2021008).

## Conflict of Interest

The authors declare that the research was conducted in the absence of any commercial or financial relationships that could be construed as a potential conflict of interest.

## Publisher’s Note

All claims expressed in this article are solely those of the authors and do not necessarily represent those of their affiliated organizations, or those of the publisher, the editors and the reviewers. Any product that may be evaluated in this article, or claim that may be made by its manufacturer, is not guaranteed or endorsed by the publisher.

## Abbreviations

MAFLD, metabolic dysfunction-associated fatty liver disease; NAFLD, nonalcoholic fatty liver disease; CT, computed tomography; MRI, magnetic resonance imaging; HSI, hepatic steatosis index; WC, waist circumference; BMI, body mass index; SBP, systolic blood pressure; DBP, diastolic blood pressure; FBG, fasting blood glucose; TC, total cholesterol; TG, triglycerides; LDL-C, low-density lipoprotein cholesterol; HDL-C, high-density lipoprotein cholesterol; ALT, alanine aminotransferase; AST, aspartate transaminase; BUN, blood urea nitrogen; SCR, serum creatinine; SUA, serum uric acid; T2DM, type 2 diabetes mellitus; HbA1c, glycosylated hemoglobin A1c; IQR, inter-quartile range; RMR, root mean square residual; CFI, comparative fitness index; OR, odds ratio; CI, confidence interval; HR, hazard ratio; NLRP3, NOD-like receptor family pyrin domain containing 3; NADPH, nicotinamide adenine dinucleotide phosphate; ROS, reactive oxygen species; IR, insulin resistance.
