# Supplementary material for: A Bidirectional Relationship Between Hyperuricemia and Metabolic Dysfunction-Associated Fatty Liver Disease
Source: Front Endocrinol (Lausanne). 2022 Feb 16;13:821689. doi: 10.3389/fendo.2022.821689 (PMC8889101; doi:10.3389/fendo.2022.821689)
Supplement: Supplementary file 1 [file DataSheet_1.docx]

**Supplementary Table 1** Baseline characteristics of 8,045 participants stratified by MAFLD in the analysis of baseline hyperuricemia and subsequent incidence of MAFLD

**Supplementary Table 2** Baseline characteristics of 13,826 participants stratified by hyperuricemia in the analysis of baseline MAFLD and subsequent incidence of hyperuricemia

**Supplementary Table 3** The cross-lagged analysis for sequential relationship between hyperuricemia and MAFLD in the sensitivity analysis 1

**Supplementary Table 4** The association between hyperuricemia and MAFLD in cohorts by Cox regression analysis.

**Supplementary Table 5** The cross-lag analysis for the sequential relationship between hyperuricemia and MAFLD

**Supplementary Table 1 Baseline characteristics of 8,045 participants stratified by MAFLD in the analysis of baseline hyperuricemia and subsequent incidence of MAFLD**

|  | **Overall**  **(n=8,045)** | **Non-MAFLD**  **(n=6,411)** | **MAFLD**  **(n=1,634)** | **P-value^*^** |
| --- | --- | --- | --- | --- |
| Age, median (IQR) years | 46.00 [37.00, 55.00] | 45.00 [36.00, 55.00] | 47.00 [39.00, 55.00] | 0.009 |
| Male sex, n(%) | 4542 (56.46) | 3411 (53.21) | 1131 (69.22) | <0.001 |
| FBG (mmol/L, median (IQR)) | 5.06 [4.73, 5.45] | 5.04 [4.72, 5.43] | 5.13 [4.79, 5.56] | <0.001 |
| LDL-C (mmol/L, median (IQR)) | 2.71 [2.27, 3.24] | 2.66 [2.24, 3.19] | 2.85 [2.39, 3.35] | <0.001 |
| HDL-C(mmol/L, median(IQR)) | 1.32 [1.12, 1.53] | 1.35 [1.16, 1.56] | 1.19 [1.04, 1.38] | <0.001 |
| TC (mmol/L, median (IQR)) | 4.63 [4.09, 5.19] | 4.61 [4.09, 5.18] | 4.67 [4.12, 5.25] | 0.010 |
| TG (mmol/L, median (IQR)) | 1.08 [0.78, 1.51] | 1.02 [0.75, 1.43] | 1.33 [0.96, 1.84] | <0.001 |
| BMI (kg/m^2^, median (IQR)) | 23.63 [21.88, 25.22] | 23.39 [21.45, 24.92] | 24.63 [23.34, 26.19] | <0.001 |
| WC(cm, median (IQR)) | 80.00 [74.00, 86.00] | 78.00 [73.00, 84.00] | 86.00 [80.00, 90.00] | <0.001 |
| ALT(U/L, median (IQR)) | 18.00 [14.00, 25.00] | 18.00 [13.00, 24.00] | 21.00 [15.80, 28.00] | <0.001 |
| AST(U/L, median (IQR)) | 21.00 [17.00, 25.00] | 20.90 [17.00, 25.00] | 21.00 [17.00, 25.00] | 0.875 |
| BUN (mmol/L, median (IQR)) | 4.86 [4.09, 5.75] | 4.81 [4.02, 5.74] | 5.00 [4.28, 5.80] | <0.001 |
| SUA (mmol/L, median (IQR)) | 307.10 [253.00, 363.00] | 300.00 [248.00, 354.00] | 333.80 [280.00, 391.00] | <0.001 |
| SCR (umol/L, median (IQR)) | 71.00 [60.00, 82.60] | 70.30 [59.00, 82.00] | 74.00 [63.05, 84.00] | <0.001 |
| SBP(mmHg, median (IQR)) | 110.00 [100.00, 125.00] | 110.00 [100.00, 124.00] | 117.00 [106.00, 130.00] | <0.001 |
| DBP (mmHg, median (IQR)) | 70.00 [68.00, 80.00] | 70.00 [67.00, 80.00] | 75.00 [70.00, 81.00] | <0.001 |
| BMI≥23, n(%) | 5056 (62.85) | 3749 (58.48) | 1307 (79.99) | <0.001 |
| Hypertension, n(%) | 1214 (15.25) | 905 (14.26) | 309 (19.13) | <0.001 |
| Diabetes, n(%) | 320 (3.98) | 228 (3.56) | 92 (5.63) | <0.001 |
| Hyperuricemia, n(%) | 554 (6.89) | 347 (5.41) | 207 (12.67) | <0.001 |

**Abbreviations:** FBG, fasting blood glucose; LDL-C, low-density lipoprotein cholesterol; HDL-C, high-density lipoprotein cholesterol; TC, total cholesterol; TG, triglycerides; WC, waist circumference; BMI, body mass index; ALT, alanine aminotransferase; AST, aspartate transaminase; BUN, blood urea nitrogen; SUA, serum uric acid; SCR, serum creatinine; SBP, systolic blood pressure; DBP, diastolic blood pressure; MAFLD, metabolic dysfunction-associated fatty liver disease.

*: P-value was calculated by Kruskal-Wallis test for continuous variables, as well as the χ2 test or Fisher's exact test for categorical variables.

**Supplementary Table 2 Baseline characteristics of 13,826 participants stratified by hyperuricemia in the analysis of baseline MAFLD and subsequent incidence of hyperuricemia**

|  | **Overall**  **(n=13,826)** | **Non-hyperuricemia**  **(n=12,310)** | **Hyperuricemia**  **(n=1,516)** | **P-value^*^** |
| --- | --- | --- | --- | --- |
| Age, median (IQR) years | 47.00 [40.00, 55.00] | 47.00 [40.00, 55.00] | 46.00 [36.00, 55.00] | <0.001 |
| Male sex, n(%) | 8851 (64.02) | 7521 (61.10) | 1330 (87.73) | <0.001 |
| FBG (mmol/L, median (IQR)) | 5.22 [4.84, 5.70] | 5.22 [4.84, 5.71] | 5.21 [4.82, 5.70] | 0.440 |
| LDL-C (mmol/L, median (IQR)) | 2.87 [2.37, 3.40] | 2.87 [2.38, 3.41] | 2.81 [2.36, 3.28] | 0.003 |
| HDL-C (mmol/L, median (IQR)) | 1.21 [1.03, 1.43] | 1.22 [1.04, 1.44] | 1.11 [0.96, 1.27] | <0.001 |
| TC (mmol/L, median (IQR)) | 4.76 [4.19, 5.35] | 4.76 [4.19, 5.36] | 4.75 [4.17, 5.31] | 0.269 |
| TG (mmol/L, median (IQR)) | 1.34 [0.92, 1.97] | 1.31 [0.90, 1.92] | 1.60 [1.12, 2.35] | <0.001 |
| WC (cm, median (IQR)) | 88.00 [80.00, 94.00] | 87.00 [80.00, 94.00] | 92.00 [86.00, 98.00] | <0.001 |
| BMI (kg/m^2^, median (IQR)) | 24.90 [23.18, 26.89] | 24.77 [23.09, 26.77] | 25.77 [24.10, 27.76] | <0.001 |
| ALT (U/L, median (IQR)) | 21.20 [15.50, 31.00] | 21.00 [15.00, 30.00] | 26.00 [19.00, 36.00] | <0.001 |
| AST (U/L, median (IQR)) | 21.00 [17.90, 26.00] | 21.00 [17.50, 26.00] | 23.00 [19.10, 28.00] | <0.001 |
| BUN (mmol/L, median (IQR)) | 4.95 [4.18, 5.80] | 4.93 [4.16, 5.80] | 5.00 [4.26, 5.83] | 0.028 |
| SCR (umol/L, median (IQR)) | 71.00 [61.00, 82.00] | 70.00 [60.00, 81.00] | 78.35 [70.00, 87.00] | <0.001 |
| SBP (mmHg,median (IQR)) | 117.00 [105.00, 130.00] | 116.00 [105.00, 130.00] | 120.00 [110.00, 131.00] | <0.001 |
| DBP (mmHg,median (IQR)) | 75.00 [70.00, 82.00] | 75.00 [70.00, 81.00] | 78.00 [70.00, 84.00] | <0.001 |
| BMI≥23, n(%) | 10723 (77.56) | 9375 (76.16) | 1348 (88.92) | <0.001 |
| Hypertension, n(%) | 2810 (20.56) | 2425 (19.91) | 385 (25.86) | <0.001 |
| Diabetes, n(%) | 1209 (8.74) | 1098 (8.92) | 111 (7.32) | 0.042 |
| MAFLD, n(%) | 6335 (45.82) | 5452 (44.29) | 883 (58.25) | <0.001 |

**Abbreviations:** FBG, fasting blood glucose; LDL-C, low-density lipoprotein cholesterol; HDL-C, high-density lipoprotein cholesterol; TC, total cholesterol; TG, triglycerides; WC, waist circumference; BMI, body mass index; ALT, alanine aminotransferase; AST, aspartate transaminase; BUN, blood urea nitrogen; SCR, serum creatinine; SBP, systolic blood pressure; DBP, diastolic blood pressure; MAFLD, metabolic dysfunction-associated fatty liver disease.

*: P-value was calculated by Kruskal-Wallis test for continuous variables, as well as the χ2 test or Fisher's exact test for categorical variables.

**Supplementary Table 3 The cross-lagged analysis for sequential relationship between hyperuricemia and MAFLD in the sensitivity analysis 1**

|  | **β1**  **(hyperuricemia to subsequent MAFLD)** | **P-value** | **β2**  **(MAFLD to subsequent hyperuricemia)** | **P-value** | **RMR** | **CFI** |
| --- | --- | --- | --- | --- | --- | --- |
| Model 1 | 0.035 (0.023, 0.047) | <0.001 | 0.025 (0.012, 0.038) | <0.001 | 0.021 | 0.988 |

**Abbreviations:** MAFLD, metabolic dysfunction-associated fatty liver disease; RMR, root mean square residual; CFI, comparative fitness index.

Model 1: adjusted age, sex, low-density lipoprotein cholesterol (LDL-C), fasting blood glucose (FBG), alanine aminotransferase (ALT), blood urea nitrogen (BUN), systolic blood pressure (SBP), triglycerides (TG), serum creatinine (SCR), high-density lipoprotein cholesterol (HDL-C).

**Supplementary Table 4** **The association between hyperuricemia and MAFLD in cohorts by Cox regression analysis.**

| **Baseline hyperuricemia to subsequent MAFLD** | | |  | **Baseline MAFLD to subsequent hyperuricemia** | | |
| --- | --- | --- | --- | --- | --- | --- |
|  | HR(95%CI) | P-value |  |  | HR(95%CI) | P-value |
| Crude | 2.669 (2.306,3.088) | <0.001 |  | Crude | 1.765 (1.593,1.955) | <0.001 |
| Model 1 | 2.191 (1.886,2.545) | <0.001 |  | Model 1 | 1.395 (1.256,1.549) | <0.001 |
| Model 2 | 1.765 (1.512,2.061) | <0.001 |  | Model 2 | 1.255 (1.116,1.412) | <0.001 |

**Abbreviations:** MAFLD, metabolic dysfunction-associated fatty liver disease.

Model 1: adjusted for age and sex.

Model 2: adjusted for age, sex, low-density lipoprotein cholesterol (LDL-C), fasting blood glucose (FBG), alanine aminotransferase (ALT), blood urea nitrogen (BUN), systolic blood pressure (SBP), triglycerides (TG).

**Supplementary Table 5 The cross-lagged analysis for the** **sequential relationship between hyperuricemia and MAFLD**

|  | **β1**  **(hyperuricemia to subsequent MAFLD)** | **P-value** | **β2**  **(MAFLD to subsequent hyperuricemia)** | **P-value** | **RMR** | **CFI** |
| --- | --- | --- | --- | --- | --- | --- |
| Model 1 | 0.039 (0.027, 0.052) | <0.001 | 0.032 (0.019, 0.045) | <0.001 | 0.021 | 0.988 |

**Abbreviations:** MAFLD, metabolic dysfunction-associated fatty liver disease; RMR, root mean square residual; CFI, comparative fitness index.

Model 1: adjusted age, sex, low-density lipoprotein cholesterol (LDL-C), fasting blood glucose(FBG), alanine aminotransferase (ALT), blood urea nitrogen (BUN), systolic blood pressure (SBP), triglycerides (TG).
